# Targeted Phototherapy for Malignant Pleural Mesothelioma: Near-Infrared Photoimmunotherapy Targeting Podoplanin

**DOI:** 10.3390/cells9041019

**Published:** 2020-04-20

**Authors:** Yuko Nishinaga, Kazuhide Sato, Hirotoshi Yasui, Shunichi Taki, Kazuomi Takahashi, Misae Shimizu, Rena Endo, Chiaki Koike, Noriko Kuramoto, Shota Nakamura, Takayuki Fukui, Hiroshi Yukawa, Yoshinobu Baba, Mika K. Kaneko, Toyofumi F. Chen-Yoshikawa, Hisataka Kobayashi, Yukinari Kato, Yoshinori Hasegawa

**Affiliations:** 1 Respiratory Medicine, Nagoya University Graduate School of Medicine, Nagoya 466-8550, Japan; 2 B3-Unit, Advanced Analytical and Diagnostic Imaging Center (AADIC)/Medical Engineering Unit (MEU), Nagoya University Institute for Advanced Research, Nagoya 466-8550, Japan; 3 Nagoya University Institute for Advanced Research, S-YLC, Nagoya 464-8601, Japan; 4 Department of Thoracic Surgery, Nagoya University Graduate School of Medicine, Nagoya 466-8550, Japan; 5 Institute of Nano-Life-Systems, Institutes of Innovation for Future Society, Nagoya University, Nagoya 464-8601, Japan; 6 Department of Biomolecular Engineering, Nagoya University Graduate School of Engineering, Nagoya 464-8601, Japan; 7 Department of Antibody Drug Development, Tohoku University Graduate School of Medicine, Sendai 980-8575, Japan; 8 Molecular Imaging Program, Center for Cancer Research, National Cancer Institute, National Institutes of Health, Bethesda, MD 20892-1088, USA; 9 New Industry Creation Hatchery Center, Tohoku University, Sendai 980-8575, Japan; 10 National Hospital Organization, Nagoya Medical Center, Nagoya 460-0001, Japan

**Keywords:** podoplanin (PDPN), near-infrared photoimmunotherapy, malignant pleural mesothelioma

## Abstract

Malignant pleural mesothelioma (MPM) has extremely limited treatment despite a poor prognosis. Moreover, molecular targeted therapy for MPM has not yet been implemented; thus, a new targeted therapy is highly desirable. Near-infrared photoimmunotherapy (NIR-PIT) is a recently developed cancer therapy that combines the specificity of antibodies for targeting tumors with toxicity induced by the photoabsorber after exposure to NIR-light. In this study, we developed a new phototherapy targeting podoplanin (PDPN) for MPM with the use of both NIR-PIT and an anti-PDPN antibody, NZ-1. An antibody–photosensitizer conjugate consisting of NZ-1 and phthalocyanine dye was synthesized. In vitro NIR-PIT-induced cytotoxicity was measured with both dead cell staining and luciferase activity on various MPM cell lines. In vivo NIR-PIT was examined in both the flank tumor and orthotopic mouse model with in vivo real-time imaging. In vitro NIR-PIT-induced cytotoxicity was NIR-light dose dependent. In vivo NIR-PIT led to significant reduction in both tumor volume and luciferase activity in a flank model (*p* < 0.05, NIR-PIT group versus NZ-1-IR700 group). The PDPN-targeted NIR-PIT resulted in a significant antitumor effect in an MPM orthotopic mouse model (*p* < 0.05, NIR-PIT group versus NZ-1-IR700 group). This study suggests that PDPN-targeted NIR-PIT could be a new promising treatment for MPM.

## 1. Introduction

Malignant pleural mesothelioma (MPM) is a malignant tumor that originates from mesothelial cells and has an extremely poor prognosis [1,2,3,4]. Although MPM is rarer compared to lung cancer, the number of patients worldwide is predicted to increase in the next decades [5,6,7]. Malignant pleural mesothelioma seldom causes metastasis to other organs but has already progressed widely at the time of diagnosis, and radical surgery is often impossible. However, the clinically available therapies for MPM are extremely limited with few regimens [3,4,8]. Therefore, the development of new therapies for unresectable MPM, especially molecularly targeted ones, is highly desirable.

Podoplanin (PDPN) is a type I transmembrane glycoprotein that is expressed in lymphatic endothelial cells, type I alveolar epithelial cells, and podocytes of the glomeruli [9]. Podoplanin is also upregulated in various tumors, including MPM, angiosarcomas, chondrosarcomas, osteosarcomas, germ-cell tumors, gliomas, glioblastomas, and squamous cell carcinomas (SCCs) of the skin, esophagus, and head and neck. The antibody (D2-40) for PDPN has been used for a long time as a specific pathological diagnostic marker to determine the development of MPM.

In 2006, NZ-1, a rat anti-human PDPN antibody, was established [10]. Podoplanin has a platelet aggregation-stimulating (PLAG) domain, of which the disialyl-core-1 structure at Thr52 of PLAG-3 is the center of platelet-aggregating activity. NZ-1 recognizes PLAG-2/3, while D2-40 recognizes PLAG-1/2 [11]. NZ-1 was shown to inhibit platelet aggregation activity of PDPN by inhibiting its interaction with platelets. NZ-1 has also been shown to have antibody-dependent cellular cytotoxicity (ADCC) and complement-dependent cytotoxicity (CDC) against MPM in vitro and in vivo [12]. NZ-1 has potent ADCC activity using rat natural killer (NK) cells as effector cells and CDC activity and mediates in vivo therapeutic antitumor activity against MPM in SCID mice. However, generally, only ADCC or CDC promotes limited antitumor effects, especially solid tumors, to eradicate tumors.

Near-infrared photoimmunotherapy (NIR-PIT) is a recently developed cancer photo-target therapy that combines the specificity of intravenously injected antibodies for targeting tumors with toxicity induced by photoabsorber (IR700) after exposure to NIR-light at 690 nm [13,14,15,16,17]. Once the NIR-light irradiates the tumor cells, immediate necrosis develops to eradicate the tumor without normal adjacent cells remaining intact [18]. Recently, the unique mechanism of cell death induced by NIR-PIT has been clarified, in which photochemical reactions change the hydrophilic status of antibody-IR700 into hydrophobic, resulting in the formation of antigen-antibody complexes aggregated on the membrane into acute cell rupture [19]. This unique cell death makes NIR-PIT a promising new modality for cancer therapy [20]. Presently, NIR-PIT is under the international phase III clinical trial against locoregional, recurrent head and neck SCC (HNSCC) (LUZERA-301, NCT03769506) with FDA fast-track approval and expected to be clinically licensed in a few years.

Among the organs in the human body, the lung and thoracic cavity can most effectively transmit NIR-light, because it consists of a large amount of air [21,22,23]. Thus, MPM is a potentially good target for NIR-PIT. Moreover, since MPM progresses more locally than distant metastasis to other organs, NIR-PIT could control local MPM with irradiation of the limited thoracic lesion [1].

In this study, we exploited NIR-PIT to develop a new phototherapy for MPM using NZ-1, targeting PDPN, and evaluated its clinical efficacy in a pleural disseminated tumor model.

## 2. Materials and Methods

### 2.1. Study Design

Our research objective was to establish a new treatment for MPM exploiting NIR-PIT. All in vivo experiments were conducted in compliance with the Guide for the Care and Use of Laboratory Animal Resources of Nagoya University Animal Care and Use Committee (approval #2017-29438, #2018-30096, #2019-31234, #2020-20104). The ethical board of Nagoya University, Clinical Research Committee, approved the use of materials (resected specimens) from the patients (approval #2017-0487). Patients were informed and consented to the use of the materials.

### 2.2. Immunostaining of Surgical Human Resected MPM Samples

We performed immunostaining of PDPN with resected tumors of patients who underwent surgery at Nagoya University Hospital from April 2004 to December 2015 and were pathologically diagnosed with MPM. Sections of 4 μm thickness were made from formalin-fixed, paraffin-embedded tumor samples and placed on glass slides. We used NZ-1 (rat monoclonal anti-PDPN antibody, 10 µg/mL) as primary antibodies, and immunohistochemical staining was performed using the VECTASTAIN Elite ABC Kit (rat IgG) (Vector Laboratories, Burlingame, CA, USA). Antigen retrieval was not performed. Color development was performed with DAB (ImmPACT DAB Substrate, Vector Laboratories, Burlingame, CA, USA) and hematoxylin. Podoplanin expression was evaluated at 40×, 100×, and 400× magnification with a bright field of microscope. When >10% of the tumor cells were stained, it was considered PDPN positive.

Additionally, we also performed immunostaining of PDPN with resected tumors of patients who were pathologically diagnosed with lung SCC and used NZ-1 and D2-40 (D2-40 mouse monoclonal antibody, Nichirei Biosciences Inc., Tokyo, Japan) as primary antibodies to compare the results of the staining. For D2-40, immunohistochemical staining was performed using the HRP-polymer secondary antibody (EnVision+ System HRP Labelled Polymer Anti-Mouse, Agilent, Santa Clara, CA, USA), and antigen retrieval techniques were performed using pH 6 buffer (Epitope Retrieval Solution pH 6, Leica Biosystems, Nussloch, Germany) and a pressure cooker.

### 2.3. Reagents

Water-soluble, silicon-phthalocyanine derivative IRDye 700DX NHS ester was purchased from LI-COR Biosciences (Lincoln, NE, USA). All other chemicals were of reagent grade.

### 2.4. Synthesis of IR700-Conjugated NZ-1

The NZ-1 (1 mg, 6.8 nmol) was incubated with IR700 NHS ester (LI-COR Biosciences, Lincoln, NE, USA) (60.226 µg, 30.8 nmol, 10 mmol/L in DMSO) in 0.1 mol/L Na_2_HPO_4_ (pH 8.6) at room temperature for 1 h. The mixture was purified with a Sephadex G50 column (PD-10; GE Healthcare, Piscataway, NJ, USA). The protein concentration was determined using a Coomassie Plus protein assay kit (Thermo Fisher Scientific, MA, USA) by measuring the absorption at 595 nm with spectroscopy (Novaspec Plus; GE Healthcare, IL, USA). The IR700 concentration was measured by absorption at 689 nm with spectroscopy to confirm the number of fluorophore molecules conjugated to mAb. The synthesis was controlled so that an average of three IR700 molecules were bound to a single antibody which is similar to that in other studies [24,25,26,27]. We performed SDS-PAGE as a quality control for the conjugate according to previous reports [28]. We used diluted NZ-1 as a nonconjugated control. The fluorescent bands were measured with an Odyssey Imager (LI-COR Biosciences) at a 700 nm fluorescence channel. To confirm the specific binding of NZ-1 to PDPN, an immunoblot with transient overexpression lysate of PDPN was performed. We abbreviate IR700 conjugated to NZ-1 as NZ-1-IR700. The detection of binding of NZ-1-IR700 on PDPN was evaluated with immunoblot according to previous reports [21,29].

### 2.5. Cell Lines

We examined 12 human MPM cell lines in this study. ACC-MESO-1, ACC-MESO-4, Y-MESO-9, Y-MESO-12, and Y-MESO-14 were established at the Aichi Cancer Research Center Institute [30,31]. MSTO-211H, NCI-H28, NCI-H226, NCI-H2052, NCI-H2373, and NCI-H2452 were purchased from American Type Culture Collection (ATCC). The 3T3 (mouse fibroblast) cells were also obtained from ATCC. The cdk4/hTERT-immortalized normal human bronchial epithelial cell line HBEC3 was obtained from the Hamon Center (University of Texas Southwestern Medical Center, Dallas, TX, USA).

### 2.6. Cell Culture

The PDPN, GFP, and luciferase-expressing MSTO-211H cells were established by transfecting RediFect Red-FLuc lentiviral particles (PerkinElmer, Waltham, MA, USA) and PDPN-GFP lentiviral particles (OriGene, Rockville, MD, USA). Luciferase-expressing H2373 cells were also established by transfecting RediFect Red-FLuc lentiviral particles (PerkinElmer, Waltham, MA, USA). Stable high PDPN, GFP, and luciferase expression was confirmed after >10 passages. We abbreviate PDPN, GFP, and luciferase-expressing MSTO-211H as MSTO-211H-PDPN-luc-GFP and luciferase-expressing H2373 as H2373-luc.

Cells were cultured in RPMI 1640 medium (Thermo Fisher Scientific, Rockford, IL, USA) supplemented with 10% fetal bovine serum and penicillin (100 IU/mL)–streptomycin (100 mg/mL) (Thermo Fisher Scientific) in tissue culture flasks in a humidified incubator at 37 °C in an atmosphere of 95% air and 5% carbon dioxide.

### 2.7. Flow Cytometry

To evaluate PDPN expression in cell lines, IR700 fluorescence from cells after incubation with NZ-1-IR700 was measured using a flow cytometer (Gallios, Beckman Coulter, Brea, CA, USA) and Kaluza software (Beckman Coulter). Cells (1 × 10^5^) were seeded on 12 well plates and incubated for 8 h at 37 °C. Thereafter, NZ-1-IR700 was added at a concentration of 10 μg/mL and incubated for 12 h. To validate the specific binding of the conjugated antibody, excess antibody (10 µg) was used before adding NZ-1-IR700 (Block Study). Both 3T3 and HBEC3 were incubated with CD16 antibody (10 μg/mL; CD16/CD32 monoclonal antibody (93), Thermo Fisher Scientific) for 12 h before the assay to inhibit nonspecific binding with cells and Fc lesion of NZ-1, and almost no signals were observed in these cell lines. Before flow cytometric analysis, the medium was replaced with PBS, and the cells were peeled from wells with pipetting or scraping.

### 2.8. Fluorescence Microscopy

To detect the antigen-specific localization of IR700 conjugates, fluorescence microscopy was performed (A1Rsi; Nikon Instech, Tokyo, Japan). Ten thousand cells were seeded on glass-bottomed dishes and incubated for 8 h. Then, NZ-1-IR700 was added to the culture medium at 10 μg/mL and incubated at 37 °C for 12 h. The cells were washed with PBS. Propidium iodide (PI, final concentration 2 µg/mL; Thermo Fisher Scientific) was used to detect ruptured cells, which were added 30 min before the observation. The cells were exposed to NIR-light (64 J/cm^2^), and serial microscopic images were obtained.

### 2.9. In Vitro NIR-PIT

One hundred thousand cells were seeded on 12 well plates and incubated with NZ-1-IR700 at 10 μg/mL for 12 h at 37 °C. After the medium was replaced with PBS, the cells were irradiated with an NIR-light-emitting diode, which emitted light at 670–710 nm wavelength (L690-66-60, Ushio-Epitex, Kyoto, Japan). The actual power density (mW/cm^2^) was measured with an optical power meter (PM100, Thorlabs, Newton, NJ, USA).

### 2.10. Cytotoxicity or Phototoxicity Assay

The cytotoxic effects of NIR-PIT with NZ-1-IR700 were determined by luciferase activity and flow cytometry with PI staining. For luciferase activity, 150 μg/mL of D-luciferin-containing media (GoldBio, St. Louis, MO, USA) was administered to PBS-washed cells 24 h after NIR-PIT and analyzed with a plate reader for detection of their bioluminescence (Powerscan4, BioTek, Winooski, VT, USA). We evaluated luciferase activity in vitro after NIR-PIT with a few timepoints (1, 6, and 24 h). With this estimation, we decided to evaluate luciferase activity in vitro at 24 h after irradiation.

For the flow cytometric assay, the cells were peeled with pipetting or scraping at 1 h after treatment and washed with PBS. The PI was added to the cell suspension (final 2 μg/mL) and incubated at room temperature for 30 min before flow cytometry. The PI fluorescence was evaluated on ten thousand cells with FACS Calibur (Becton Dickinson, Franklin Lakes, NJ, USA).

### 2.11. Animal and Tumor Models

All in vivo procedures were conducted in compliance with the local Animal Care and Use Committee of Nagoya University. Eight-to-twelve-week-old female homozygote athymic nude mice were purchased from Chubu Kagaku Shizai (Nagoya, Japan). During the procedures, the mice were anesthetized with isoflurane.

To examine the distribution of NZ-1-IR700 in vivo, we prepared a xenograft tumor mouse model of MSTO-211H-PDPN-luc-GFP in the right dorsum. Six million MSTO-211H-PDPN-luc-GFP cells were injected subcutaneously into the right dorsum of the mice. The mice received 100 μg of NZ-1-IR700 intravenously via the tail vein at 14 days after cell implantation and analyzed with a fluorescence imager (Pearl Imager, LI-COR Biosciences) for detecting IR700 fluorescence.

Six million MSTO-211H-PDPN-luc-GFP and H2373-luc cells were injected subcutaneously into the dorsum of the mice. The greatest longitudinal diameter (length) and greatest transverse diameter (width) were measured with an external caliper. Tumor volumes based on caliper measurements were calculated using the following formula: tumor volume = length × width^2^ × 0.5. Tumors that reached approximately 100 mm^3^ in volume were selected for further experiments. Body weight was checked on a scale. For bioluminescence imaging (BLI), D-luciferin (15 mg/mL, 200 μL) was injected intraperitoneally into mice 13 days after cell implantation and analyzed with IVIS imaging system (PerkinElmer) for luciferase activity. Then, mice were selected for further study based on tumor size and bioluminescence signals. The mice were sacrificed with carbon dioxide when the tumor length was >15 mm.

To create a pleural disseminated MPM model, six million MSTO-211H-PDPN-luc-GFP or one hundred thousand cells H2373-luc cells in PBS were injected into the thoracic cavity through the right intercostal space using a 30 G needle according to a previous study [14]. To prevent lung injury, the needle could only be inserted 5 mm (a foam styrol stopper prevented deeper insertion). After 4 days, BLI was performed after D-luciferin (15 mg/mL, 200 μL) was injected intraperitoneally, and mice with sufficient luciferase activity were selected for further studies.

### 2.12. Characterization of Mouse Model

Both the subcutaneous bilateral flank model and disseminated pleural model received 100 μg of NZ-1-IR700. One day after injection, serial images were obtained with a fluorescence imager (Pearl Imager, LI-COR Biosciences) for detecting IR700 fluorescence with the IVIS imaging system for BLI.

### 2.13. In Vivo Local PDPN-Targeted NIR-PIT

The day when the tumor cells were injected into the mice was indicated as day 1. The mice with tumors on both dorsal sides were injected with 100 μg of NZ-1-IR700 on day 13, and larger tumors were irradiated with NIR-light at 50 J/cm^2^ on day 14 and 100 J/cm^2^ on day 15.

For evaluation of NIR-PIT effects in the pleural disseminated MPM mouse model, the mice were injected with 100 μg of NZ-1-IR700 on day 4 and administered at 30 J/cm^2^ on day 5. The NIR-light was applied 15 J/cm^2^ from two directions transcutaneously followed by serial fluorescence imaging (FLI) and BLI.

### 2.14. Statistical Analysis

Data are expressed as mean ± SEM from a minimum of three experiments, unless otherwise indicated. Statistical analyses were performed using a statistical program (GraphPad Prism, GraphPad Software, San Diego, CA, USA). For two-group comparisons, the Mann–Whitney test or *t*-test was used. *p*-Value < 0.05 indicated a statistically significant difference.

## 3. Results

### 3.1. Immunostaining of Surgical Specimens of Human-Resected MPM

**Figure 1:**
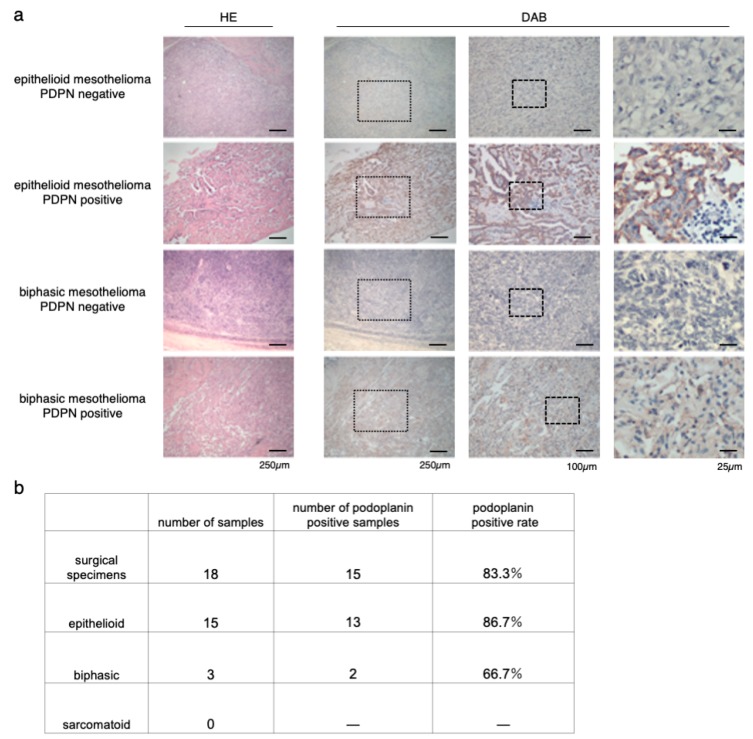
Immunostaining of surgical specimens of human resected MPM (malignant pleural mesothelioma). (**a**) Hematoxylin-eosin (HE) and podoplanin (PDPN) staining of epithelioid and biphasic MPM specimens. Representative images of both PDPN-positive and PDPN-negative MPM specimens are shown. The medium column showed the magnified view of the dot frame in left column. The right side column showed the magnified view of the dot frame in the medium column. (**b**) Number and rate of PDPN-positive specimens in each MPM type.

To confirm the coincidence of the staining between NZ-1 and D2-40, we first checked both staining on the same specimens, and no staining difference was detected (Supplementary Materials Figure S1). Immunostaining for PDPN with NZ-1 was performed on 18 surgical specimens of MPM in our institution (Figure 1a). Of 18 patients, 15 had epithelioid mesothelioma, and three had biphasic mesothelioma. Sarcomatoid mesothelioma was excluded. The positive rate of PDPN staining was 86.7% (13/15) in epithelioid mesothelioma and 66.7% (2/3) in biphasic mesothelioma. The positive rate of all cases across the tumor type was 83.3% (15/18) (Figure 1b). This result was almost consistent with those of previous studies [32,33,34].

### 3.2. Conjugation of NZ-1 with IR700DX

**Figure 2:**
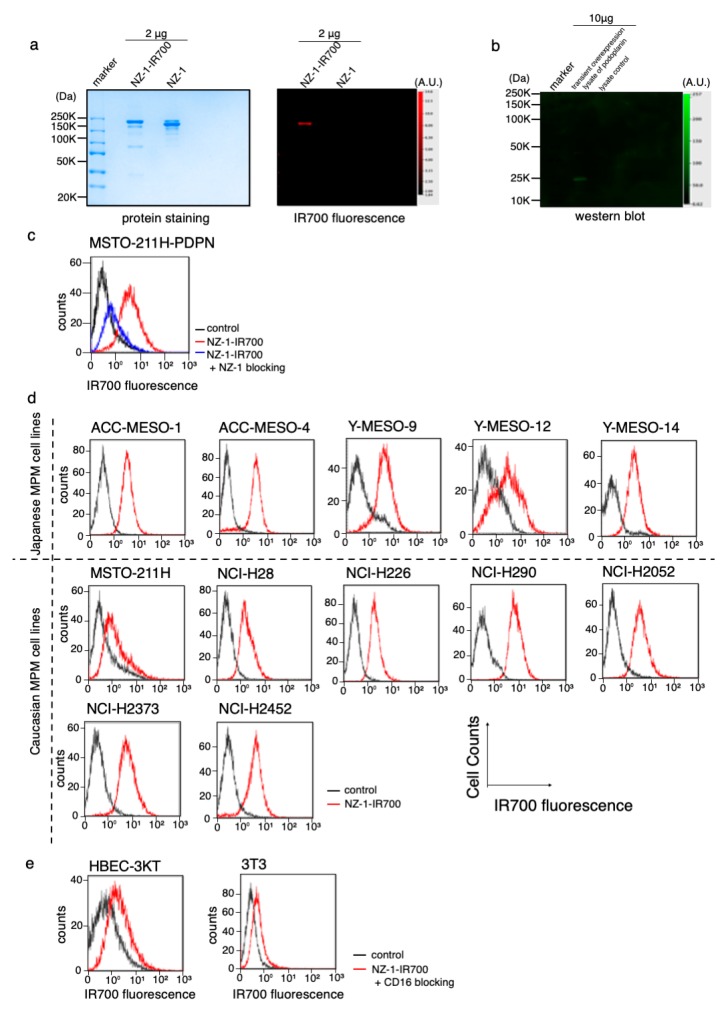
Conjugation of NZ-1-IR700 and its binding capacity. (**a**) Successful confirmation of conjugated NZ-1-IR700 with SDS-PAGE (left, colloidal blue staining; right, fluorescence at 700 nm channel). Diluted NZ-1 served as a control. (**b**) Immunoblotting with NZ-1-IR700 in PDPN-overexpressing cell lysates. Specific binding of NZ-1-IR700 to PDPDN was detected. (**c**) Prior incubation with excess NZ-1 (to block PDPN) inhibited the binding of NZ-1-IR700 to MSTO-211H-PDPN cells, indicating that NA-1-IR700 binds specifically to PDPN. (**d**) PDPN expression in various MPM cell lines. Examination of PDPN expression in MPM cell lines established from Caucasian and Japanese patients with MPM. Almost all MPM cell lines, except for Y-MESO-12 and MSTO-211H, expressed PDPN and showed IR700 fluorescence. (**e**) Flow cytometric analysis of NZ-1-IR700 in HBEC (normal bronchial epithelial cells) and mouse fibroblast 3T3 cells. Unspecific binding to normal or mouse fibroblasts was not detected.

The NZ-1-IR700 was qualified with the 700 nm fluorescence signals by SDS-PAGE (Figure 2a). The specific binding of NZ-1-IR700 to PDPN was evaluated by immunoblotting with a transiently overexpressed PDPN cell lysate (Figure 2b). Blocking of signals on MSTO-211H-PDPN with the addition of excess NZ-1 was confirmed (Figure 2c), suggesting that NZ-1-IR700 bound specifically on PDPN. The NZ-1-IR700 did not bind to 3T3 cells (mouse fibroblasts) or human normal tracheal epithelia, HBEC3 (Figure 2e). Collectively, NZ-1-IR700 was successfully produced and specifically binds to PDPN.

### 3.3. PDPN Expression in the MPM Cell Lines

To evaluate PDPN expression in a variety of MPM cell lines, IR700 fluorescence signals were detected with NZ-1-IR700 on ACC-MESO-1, ACC-MESO-4, Y-MESO-9, Y-MESO-12, and Y-MESO-14 (Japanese MPM cell lines) and MSTO-211H, NCI-H28, NCI-H226, NCI-H290, NCI-H2052, NCI-H2373, and NCI-H2452 (Caucasian MPM cell lines) using a flow cytometer. Most MPM cell lines, except for Y-MESO-12 and MSTO-211H, expressed PDPN and showed IR700 fluorescence (Figure 2d).

These data suggest that PDPN is widely expressed in MPM cell lines across races.

### 3.4. In Vitro Microscopic Observation of NIR-PIT with NZ-1-IR700 on MPM Cell Lines

To optically monitor the effect of NIR-PIT, we genetically modified MSTO-211H (PDPN-negative cells) to express luciferase and PDPN; MSTO-211H-PDPN-luc-GFP and H2373 to express luciferase; H2373-luc (Supplementary Materials Figure S2).

**Figure 3:**
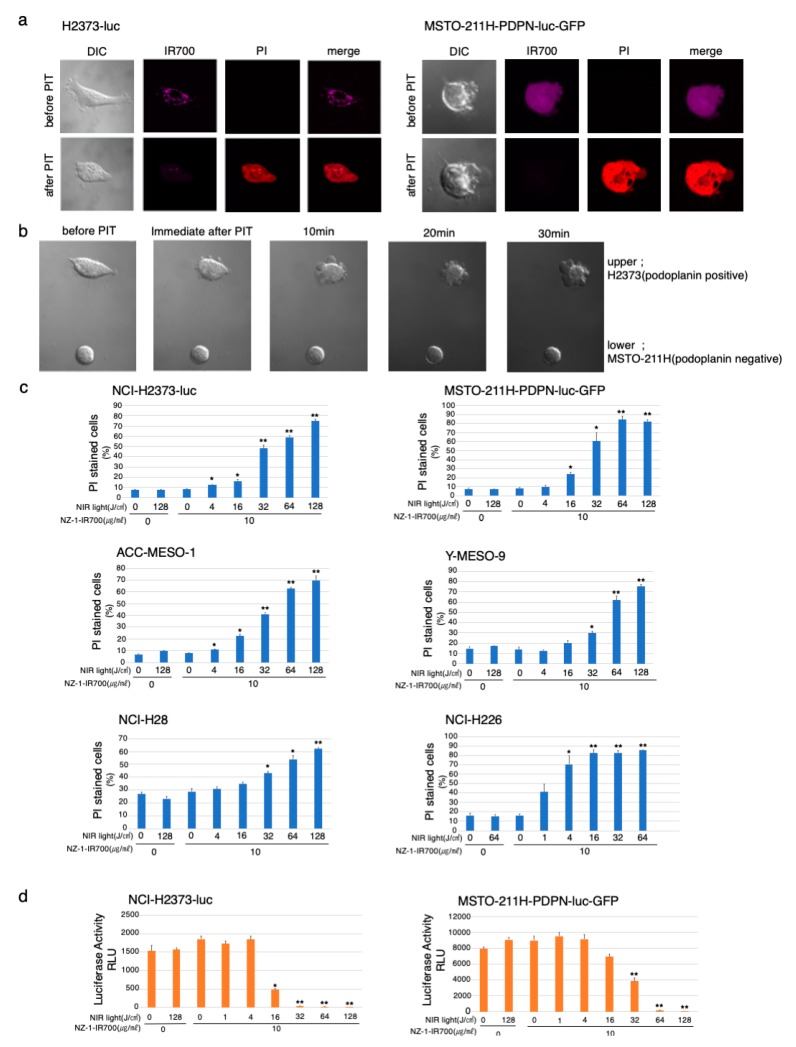
In vitro effects of NIR-PIT with NZ-1-IR700 on MPM cell lines. (**a**) Microscopic observations before and after PDPN-targeted NIR-PIT on H2373-luc and PDPN-overexpressing MSTO-211H cells with NIR-light (16 J/cm^2^). (**b**) Co-cultured H2373 (PDPN positive) and MSTO-211H (PDPN negative) were used to assess the selective effect of NIR-PIT with NIR-light (16 J/cm^2^). Only PDPN-expressing H2373 cells ruptured after the therapy. (**c**) Membrane damage induced by PDPN-targeted NIR-PIT was measured with the dead cell count using PI staining with flow cytometer which increased in a manner dependent on the light dose. Data are presented as means ± SEM (*n* ≥ 4, * *p* < 0.05, ** *p* < 0.01, Student’s *t*-test). The cell death effect of PDPN-targeted NIR-PIT was universally demonstrated across the MPM cell lines. (**d**) Luciferase activity in H2373-luc and MSTO-211H-PDPN-luc-GFP cells was measured as relative light unit (RLU) which also decreased in an NIR-light dose-dependent manner. Data are presented as means ± SEM (*n* ≥ 4, *p < 0.05, **p < 0.01, Student’s *t*-test).

Serial fluorescence microscopic observation of MSTO-211H-PDPN-luc-GFP and H2373-luc cells was performed before and after NIR-PIT. After NIR-light (64 J/cm^2^) irradiation of both MSTO-211H-PDPN-luc-GFP and H2373-luc cells, cellular swelling, bleb formation, and increase in the fluorescence of dead cell staining were observed (Figure 3a). Time-lapse imaging of MSTO-211H-PDPN-luc-GFP showed acute morphological changes and an increase in fluorescence of PI staining within 30 min (Supplementary Materials Video S1). A decrease in IR700 fluorescence was also observed after NIR-light exposure.

To assess specific cell death induced by NIR-PIT with NZ-1-IR700, co-cultures of H2373 (PDPN positive) and MSTO-211H (PDPN negative) were set and followed by NIR-PIT with NZ-1-IR700 (Figure 3b). The H2373 begins to swell immediately after NIR-light irradiation into cell rupture, in contrast, MSTO-211H did not change at all.

These data showed that NIR-PIT with NZ-1-IR700 could selectively destroy PDPN-expressing cancer cells without damaging nontarget cells.

### 3.5. In Vitro NIR-PIT Effects with NZ-1-IR700 in MPM Cell Lines

To quantitate in vitro NIR-PIT with NZ-1-IR700, we examined luciferase activity and PI staining with flow cytometry for various MPM cell lines (H2373-luc, MSTO-211H-PDPN-luc-GFP, ACC-MESO-1, Y-MESO-9, NCI-H28, and NCI-H226). Based on the incorporation of PI, the percentage of cell death increased in a light dose-dependent manner, regardless of the cell lines (Figure 3c). Luciferase activity showed significant decreases in relative light units (RLUs) in NIR-PIT-treated cells. The BLI also showed a decrease in luciferase activity in a light dose-dependent manner, regardless of the cell lines. No significant cytotoxicity was observed with NIR-light exposure alone or NZ-1-IR700 alone in either luciferase activity or PI staining (Figure 3d).

Collectively, these studies confirmed that NIR-PIT with NZ-1-IR700 can cause necrotic cell death, universally for MPM cancer cells with PDPN expression.

### 3.6. In Vivo Biodistribution of NZ-1-IR700

**Figure 4:**
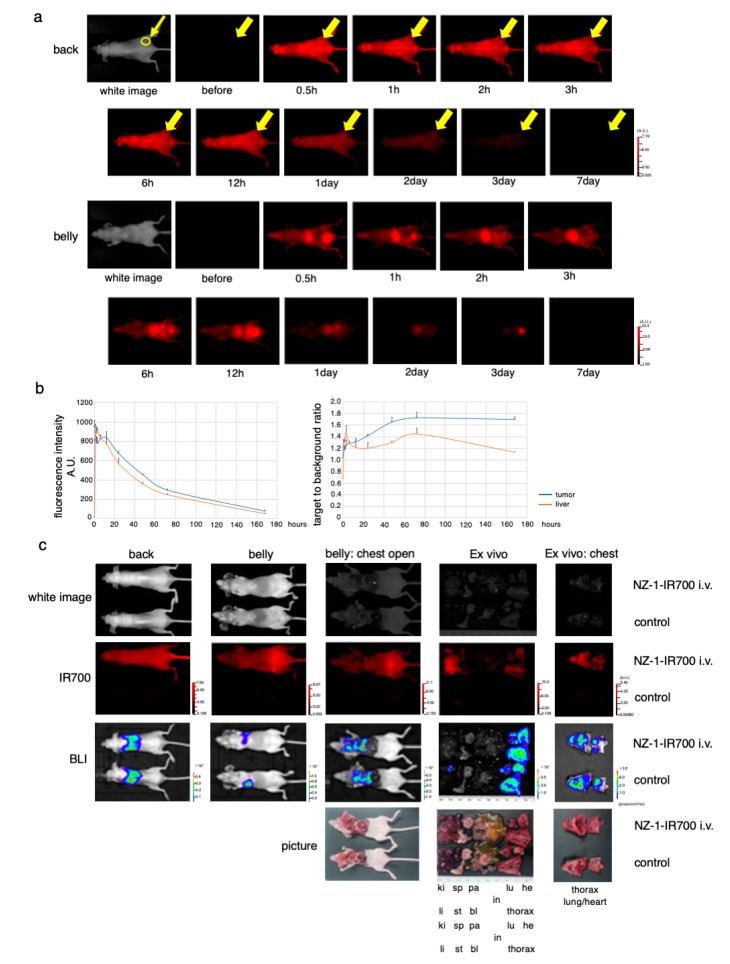
In vivo biodistribution of NZ-1-IR700. (**a**) Representative images (FLI) before and after intravenous injection of NZ-1-IR700 (fluorescence imager, 700 nm). The yellow arrow indicates the tumor site. (**b**) Fluorescence intensity measurement of the tumor and liver (left). The target-to-background ratio of the tumor and liver are indicated (right). Data are presented as mean ± SEM (*n* = 3). Fluorescence quantification showed that the highest accumulation at the tumor site was noted at day 1, while the highest target-to-background ratio (TBR) was observed on days 2 and 3. (**c**) Characterization of the pleural disseminated MPM model (MSTO-211H-PDPN-luc-GFP). Ex vivo fluorescence imaging of the disseminated pleural model at 1 day after NZ-1-IR700 injection and BLI with the IVIS imaging system (ki, kidney; sp, spleen; pa, pancreas; li, liver; st, stomach; bl, bladder; in, intestine; lu, lung; he, heart). Intravenous injection of NZ-1-IR700 was detected in disseminated tumors.

After injection of NZ-1-IR700 into the subcutaneous flank model, IR700 fluorescence was observed on the whole mouse body and gradually accumulated at the tumor site 2 h after the injection (Figure 4a). Fluorescence quantification showed that the highest accumulation at the tumor site at day 1, while the highest target-to-background ratio (TBR) was observed on days 2 and 3. There was no other specific accumulation of IR700-fluorescence, except for the liver and bladder, which involved the process of metabolism and excretion (Figure 4b).

These results suggested that NZ-1-IR700 was specifically distributed in PDPN-expressing tumors and that treatments 1 and 2 days after NZ-1-IR700 administration were suitable.

### 3.7. In Vivo Antitumor Effect of PDPN-Targeted NIR-PIT

**Figure 5:**
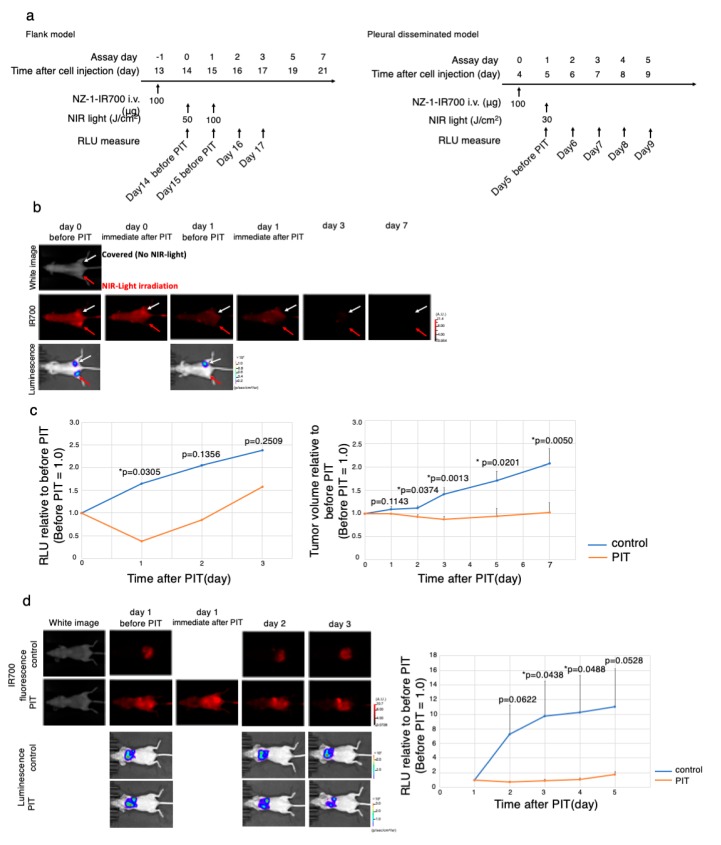
In vivo antitumor effect of PDPN-targeted NIR-PIT. (**a**) The PDPN-targeted NIR-PIT regimen is shown in a line. (**b**) In vivo FLI and BLI of subcutaneous bilateral flank xenografted mice model. Arrow showed tumor site. Red Arrow tumor was treated with NIR-PIT. (**c**) Quantitative RLU demonstrated a significant decrease in PDPN-targeted NIR-PIT-treated tumors (before NIR-PIT = 1) (* *p* < 0.05 versus control, *t*-test). PDPN-targeted NIR-PIT also leads to significant reductions in tumor volume (* *p* < 0.05 versus control, *t*-test) in a flank model (*n* = 4 mice in each group). (**d**) PDPN-targeted NIR-PIT in the pleural disseminated model. FLI and BLI of the pleural disseminated model are shown on the left. Quantitative RLUs showed that PDPN-targeted NIR-PIT led to significant reductions in luciferase activity (* *p* < 0.05 versus control, *t*-test, *n* ≥ 3 mice in each group).

The NIR-PIT regimen and imaging protocol are depicted in Figure 5a.

To monitor PDPN-targeted antitumor effects induced by NIR-PIT, BLI and FLI were examined in a mouse xenograft model with a subcutaneous bilateral flank tumor (H2373-luc, MTSO-211H-PDPN-luc-GFP) model. The IR700 fluorescence disappeared only at the treated site (right) immediately after NIR-PIT, suggesting that the therapy went well (Figure 5b, Supplementary Materials Figure S4a). The BLI showed that luminescence inside the treated tumor disappeared 1 day after NIR-PIT (* *p* < 0.05 versus no NIR-light irradiation tumor (control; NZ-1-IR700 iv only), *t*-test), indicating that the antitumor effect was strong. In contrast, the RLU of non-irradiated tumors increased along with tumor growth. Antitumor effect evaluated by both tumor volume and luciferase activity showed a significant decrease (* *p* < 0.05 versus no NIR-light irradiation (control; NZ-1-IR700 iv only), *t*-test) (Figure 5c, Supplementary Materials Figure S4b).

Therefore, PDPN-targeted NIR-PIT with NZ-1-IR700 demonstrated a significant antitumor effect on the subcutaneous flank tumor model.

### 3.8. Characterization of the Pleural Disseminated MPM Model

Next, to evaluate the PDPN-targeted NIR-PIT with NZ-1-IR700 in a more precise model, an orthotopic thoracic disseminated MPM model was established. To confirm the MPM pleural disseminated orthotopic model with both MSTO-211H-PDPN-luc-GFP and H2373-luc cells, BLI and FLI were performed. The implanted thoracic disseminated tumors with NZ-1-IR700 intravenously injected via the tail vein 1 day before imaging showed both high IR700 fluorescence and luciferase activities, indicating that intravenously injected NZ-1-IR700 accumulated on the disseminated pleural tumor sites (Figure 4c, Supplementary Materials Figure S3).

These data suggested that MPM pleural disseminated orthotopic models were successfully established and that intravenous injection of NZ-1-IR700 could reach and accumulate on the disseminated tumors.

### 3.9. In Vivo PDPN-Targeted NIR-PIT Effect on MPM Pleural Disseminated Orthotopic Model

To evaluate the effect on the MPM pleural disseminated orthotopic model with H2373-luc tumor, PDPN-targeted NIR-PIT with NZ-1-IR700 was performed. After treatment with NIR-PIT, pleural disseminated tumors in the model showed a decrease in both IR700 fluorescence and luciferase activities. While the RLU did not increase in the NIR-PIT-treated groups, RLU in the control group showed a gradual increase along with tumor growth (* *p* < 0.05 versus control, *t*-test) (Figure 5d). Collectively, these data suggested that NIR-PIT caused significant antitumor effects even in the MPM pleural disseminated orthotopic model, and the therapy was feasible.

## 4. Discussion

This study established a novel molecular targeted therapeutic approach targeting PDPN for MPM. Immunostaining of the MPM-resected specimens showed that approximately 80% of MPM specimens were positive for PDPN. We also found that a variety of MPM cell lines express PDPN across races. In vitro NIR-PIT showed high selective cytotoxicity to the various PDPN-positive MPM cell lines. We also demonstrated the antitumor effect on orthotopic MPM mice model with the combination of NZ-1 and NIR-PIT technology. The therapy is feasible and can be performed with repeated NIR-light irradiation.

The only standard chemotherapy for MPM is cisplatin + pemetrexed (CDDP + PEM) combination therapy. Bevacizumab, a humanized monoclonal antibody against vascular endothelial growth factor (VEGF), has been shown to prolong the median overall survival (OS) from 16.1 to 18.8 months and progression-free survival from 7.3 to 9.2 months when used in combination with CDDP + PEM in the phase III MAPS (NCT00651456) [35]. Additional anti-angiogenic drugs, such as cediranib (anti-VEGF receptor and platelet-derived growth factor (PDGF) receptor inhibitor), nintedanib (anti-VEGFR, PDGFR, and fibroblast growth factor (FGF) receptor inhibitor), axitinib (anti-VEGFR inhibitor), and soferanib (multi-target inhibitor of VEGFR1/2/3, FGFR-1, PDGFR-β, and RAF/cKit pathway) have also been examined for additive effects; however, they have only limited effects. Recently, the effects of immune checkpoint inhibitors (ICIs), including anti-CTLA-4 antibodies and anti-PD-1/PD-L1 antibodies, on various malignant tumors have been reported [36,37,38]. A combination of ICIs and chemotherapy has been evaluated in several clinical trials. With this progression, patients with MPM still have few options for therapy.

The development of antibodies targeting PDPN had started decades ago. NZ-1 was developed as a new anti-human PDPN antibody by immunizing rats with a PLAG domain of PDPN in 2006. NZ-1 inhibits the platelet aggregation activity of PDPN by suppressing its interaction with platelets. Many other clones targeting PDPN have also been produced and proved to show some antitumor effect based on ADCC and CDC. Since anti-human PDPN antibodies including NZ-1 also recognize normal cells such as lymphatic endothelial cells, a cancer-specific mAb (CasMab) that specifically recognizes human PDPN in tumor cells was established in 2014 [39]. When the same glycoprotein is expressed in cancer and normal cells, CasMab focuses on the difference in the type of glycosylation and recognizes both peptides and sugar chains simultaneously. With this new technology of antibody modification, more specific and effective antibodies have emerged.

PDPN has been recently reported to be expressed in not only cancer cells but also cancer-associated fibroblasts (CAFs) of various malignant tumors [9,40]. PDPN expression in CAFs is thought to be correlated with poor prognosis and associated with lymph node metastasis and reduced OS in many malignancies [41,42]. Thus, targeting PDPN has the additional advantage of depleting CAFs which is essential for tumor progression.

NIR-PIT is a selective and novel cancer treatment that is labeled with an antibody and reacts only at the site irradiated with NIR-light. Thus, the therapy can control spatiotemporal anti-cancer effects, resulting in an ideal targeted molecular therapy for patients with MPM. The results of a phase II clinical trial of NIR-PIT in patients with locoregional recurrent HNSCC were reported in American Society of Clinical Oncology 2019 (55th Annual Meeting of the American Society of Clinical Oncology, NCT02422979). The overall response rate was 43% of which four had complete response. Based on these results, a randomized, double-arm, open-label, controlled international phase III clinical trial (LUZERA-301) is now underway. This clinical trial obtained “fast-track” designation from the US FDA and “SAKIGAKE” designation from Japan Pharmaceuticals and Medical Devices Agency (PMDA), and this NIR-PIT technology is expected to be in clinical use in a few years.

The detailed cytotoxic mechanism of NIR-PIT has been unclear for several years. A recent study on the mechanism of NIR-PIT revealed that photochemical ligand reactions trigger rapid necrotic cell death. This reaction converts the hydrophilic side chains (silanol) of IR700 into hydrophobic which renders the antibody-IR700 conjugates aggregated on the cell membrane. This photochemical reaction-induced cell death by NIR-PIT provides therapy uniqueness for acute photonecrosis [19,20]. This new concept of cell death makes NIR-PIT totally different from conventional phototherapies.

There are interesting approaches to treat cancers with antibodies [43]. Firstly, the effect of radioimmunotherapy (RIT) using the rat-human chimeric antibody NZ-12 has been reported [44]. NZ-12 is applicable for RIT and radiolabeled NZ-12 enhances the antitumor effect. RIT with radiolabeled NZ-12 significantly suppressed tumor growth and prolonged survival without body weight loss and obvious adverse effects. RIT irradiates β particles (^90^Y), thus RIT has to give normal tissue damaged and the accumulation of the liver caused liver damage. Additionally, linking toxins to antibodies to generate “immunotoxins” has shown some success as an alternative cancer therapy. However, the efficacy is often dose dependent, as use of immunotoxins has been associated with adverse side effects such as liver damage [45]. Conversely, the advantage of NIR-PIT is that it is safe; phototherapy is targeted to the region of interest, and there is no toxicity associated with antibody-IR700 conjugates in the absence of NIR-light irradiation.

There are some concerns in this study. First, since PDPN is also expressed in lymphatic endothelial cells, the antibody targeting PDPN also binds to them. To avoid binding to lymphatic endothelial cells [9], CasMab, which specifically recognizes tumor cells based on the difference in the glycosylation of PDPN between tumor and normal cells, has recently emerged [39]. With the combination of NIR-PIT and CasMab technology for PDPN targeting, we could achieve ideal selective cancer cell death targeting PDPN. Second, it is difficult to irradiate the chest cavity via external light sources. However, in actual clinical use, we intend to use diffuser light sources to irradiate the entire intrathoracic cavity via the thoracic drainage tube, since we usually do pretreat to discharge the pleural effusion and place the drainage device (create a hole and place the tube into the cavity) in the chest wall [8]. Thus, we can irradiate the entire thoracic cavity via both the external and internal chest walls. With the device development in the future, this technology could overcome this difficulty.

MPM is thought to be suitable for NIR-PIT because it tends to locally invade only in the thoracic cavity and has rare distant metastases [1]. However, in some cases, NIR-PIT has limited therapeutic effects. In such cases, since the adverse events caused by NIR-PIT are negligible, NIR-PIT could be repeated or combined with the therapy used with ADCs [46,47], chemotherapy [8], immune checkpoint inhibitors [48], and anti-regulatory T-cell therapies [49].

Therefore, NIR-PIT targeting PDPN with NZ-1 showed sufficient antitumor effects and was thought to be feasible. Thus, NIR-PIT targeting PDPN could be a promising new treatment for MPM.

## Supplementary Materials

The following are available online at https://www.mdpi.com/2073-4409/9/4/1019/s1, Figure S1: Immunostaining of surgical specimens of human resected lung squamous cell carcinoma. Figure S2: Confirmation of the stable expression of luciferase. Figure S3: Characterization of the pleural disseminated MPM model (H2373-luc). Figure S4: In vivo antitumor effect of PDPN-targeted NIR-PIT on MSTO-211H-PDPN-luc-GFP tumor. Video S1: Time-lapse imaging of MSTO-211H-PDPN-luc-GFP with microscopic observations before and after NIR-PIT for 30 min.
