# Supplementary material for: Targeted Phototherapy for Malignant Pleural Mesothelioma: Near-Infrared Photoimmunotherapy Targeting Podoplanin
Source: Cells. 2020 Apr 20;9(4):1019. doi: 10.3390/cells9041019 (PMC7225918; doi:10.3390/cells9041019)
Supplement: Supplementary file 1 [file cells-09-01019-s001.zip › Supplement/Supplementary Video .pptx]

## Slide 1
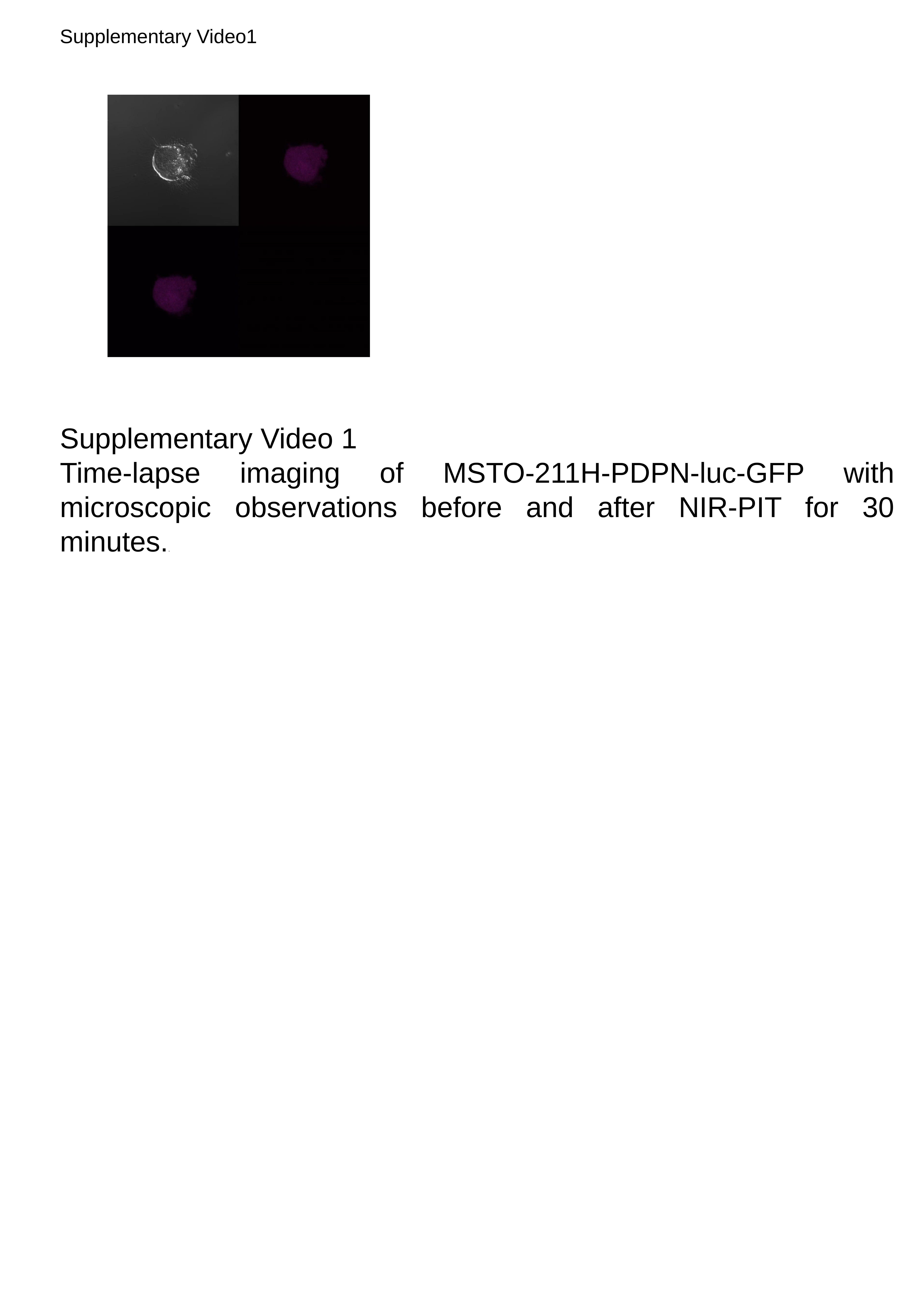

Supplementary Video1
Supplementary Video 1
Time-lapse imaging of MSTO-211H-PDPN-luc-GFP with microscopic observations before and after NIR-PIT for 30 minutes..
