# Supplementary material for: Targeted Phototherapy for Malignant Pleural Mesothelioma: Near-Infrared Photoimmunotherapy Targeting Podoplanin
Source: Cells. 2020 Apr 20;9(4):1019. doi: 10.3390/cells9041019 (PMC7225918; doi:10.3390/cells9041019)
Supplement: Supplementary file 1 [file cells-09-01019-s001.zip › Supplement/Final Supplementary Figure PDPN .pdf]

Supplementary Materials

Targeted Phototherapy for Malignant Pleural Mesothelioma: Near-Infrared Photoimmunotherapy Targeting Podoplanin

Supplementary Figure1

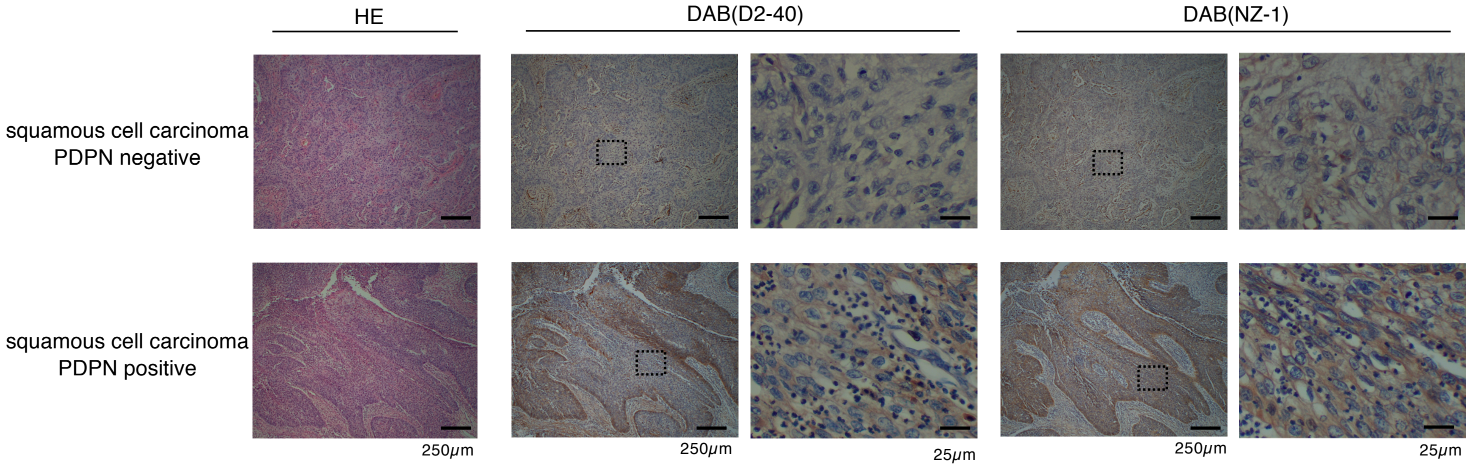

Supplementary Figure 1

Immunostaining of surgical specimens of human resected lung squamous cell carcinoma. Hematoxylin-eosin and podoplanin (PDPN) staining with D2-40 and NZ-1 in PDPN-negative and PDPN-positive squamous cell carcinoma cells. Both D2-40 and NZ-1 can stain PDPN with the same lesion in the same specimen section.

Supplementary Figure2

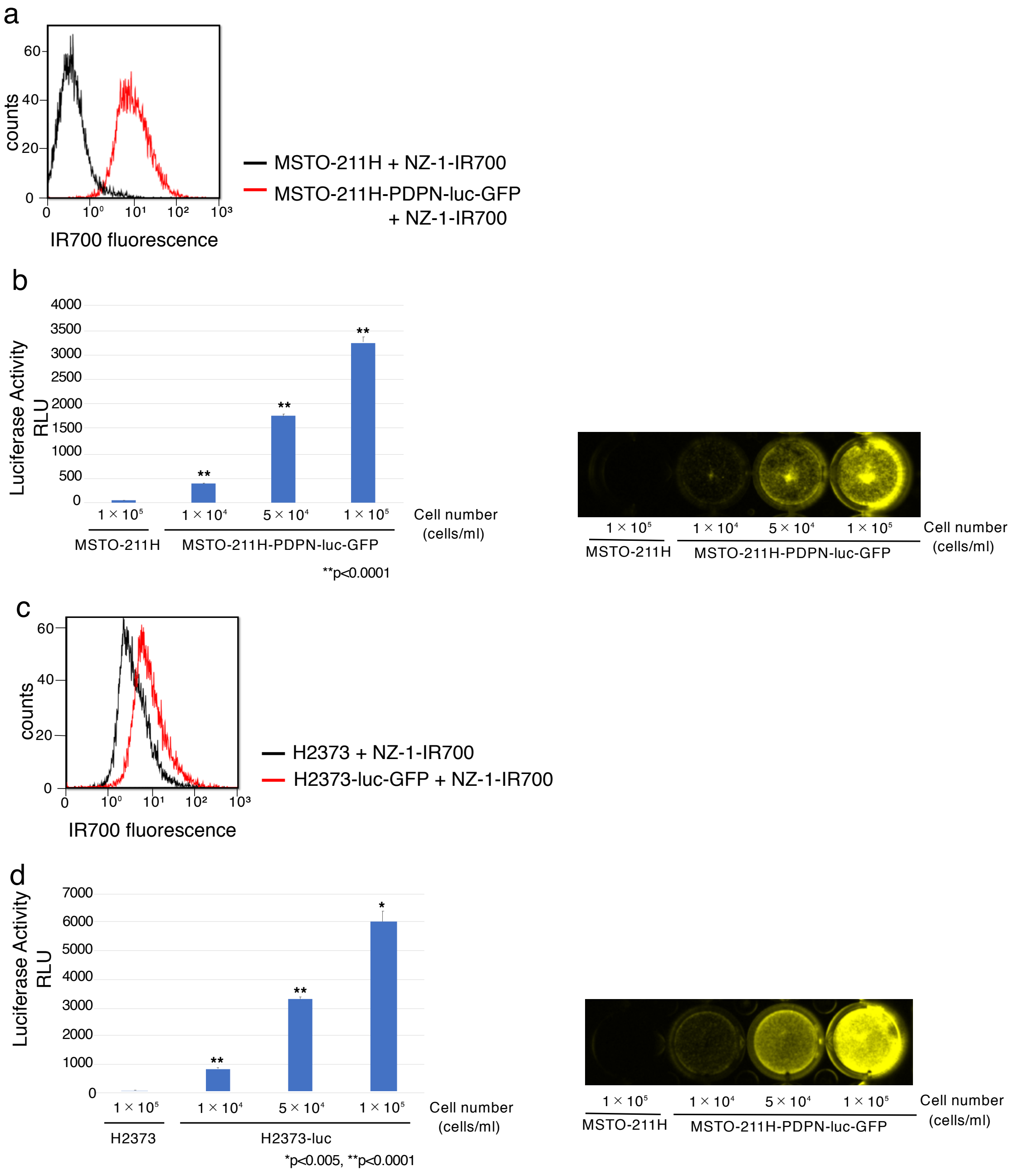

Supplementary Figure 2

Confirmation of the stable expression of luciferase.

(a) Establishment of PDPN and luciferase-expressing cell lines. Both MSTO-211H-PDPN-luc-GFP and MSTO-211H showed the same fluorescence signal level as that of flow cytometry.

(b) Luciferase expression of MSTO-211H-PDPN-luc-GFP was confirmed with a bioluminescence plate reader.

(c) Establishment of luciferase-expressing H2373 cells (H2373-luc). Both H2373-luc and H2373 showed the same fluorescence signal level as that of flow cytometry.

(d) Luciferase expression of H2373-luc was confirmed using a bioluminescence plate reader.

Supplementary Figure3

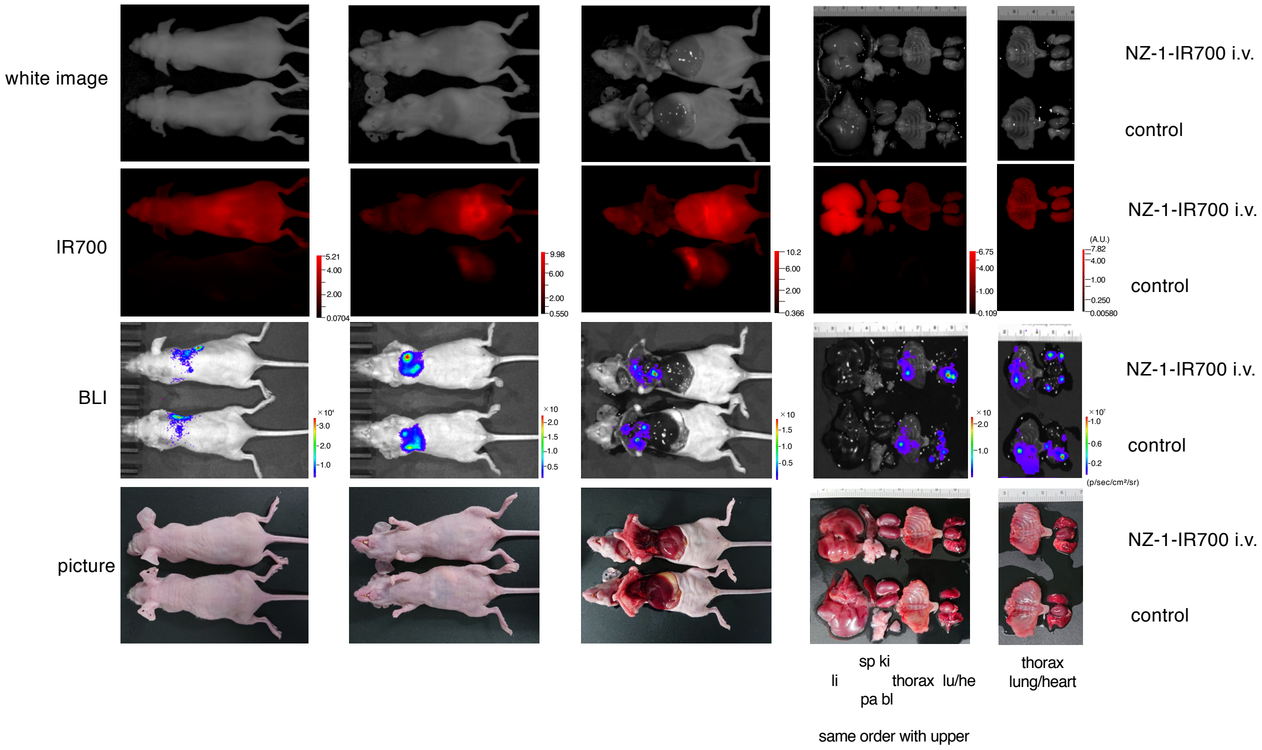

Supplementary Figure 3

Characterization of the pleural disseminated MPM model (H2373-luc). *Ex vivo* FLI of the disseminated pleural model 1 day after NZ-1-IR700 injection and BLI with the IVIS imaging system (ki, kidney; sp, spleen; pa, pancreas; li, liver; st, stomach; bl, bladder; in, intestine; lu, lung; he, heart). Intravenous injection of NZ-1-IR700 was detected in the disseminated tumors.

Supplementary Figure4

a

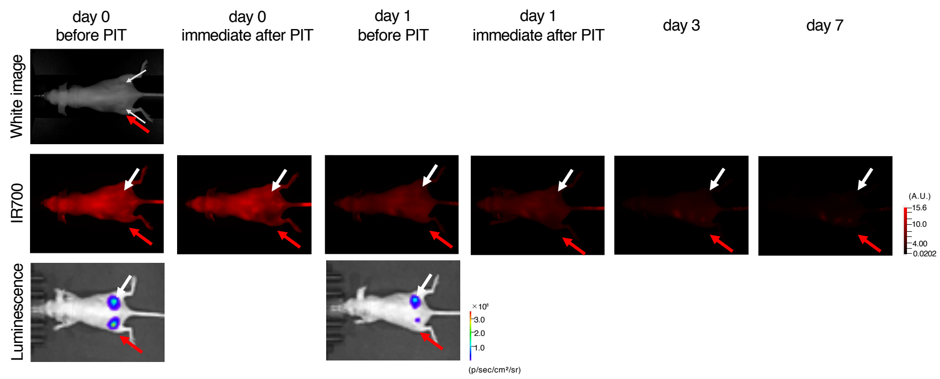

b

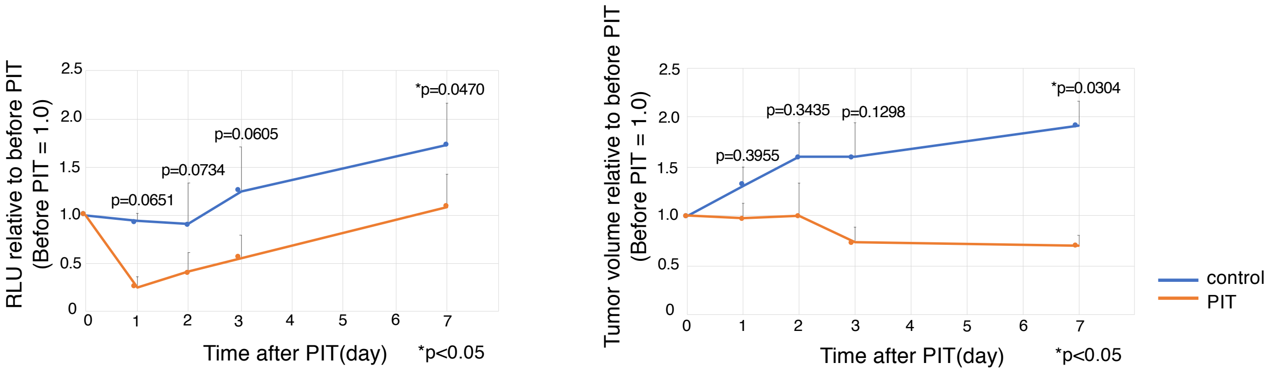

Supplementary Figure 4

In vivo antitumor effect of PDPN-targeted NIR-PIT on MSTO-211H-PDPN-luc-GFP tumor.

(a) *In vivo* FLI and BLI in the subcutaneous bilateral flank xenograft model.

(b) Quantitative RLU demonstrated a significant decrease in PDPN-targeted NIR-PIT-treated MSTO-211H-PDPN-luc-GFP tumors (before NIR-PIT=1) (*p<0.05 vs. control, t test). PDPN-targeted NIR-PIT on MSTO-211H-PDPN-luc-GFP tumors also led to significant reductions in tumor volume (*p<0.05 vs. control, t test) in a flank model (n=4 mice in each group).

Supplementary Video1

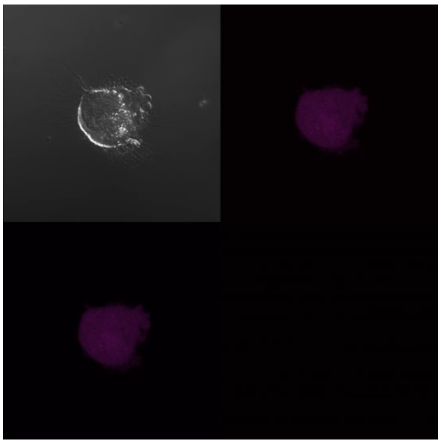

Supplementary Video 1

Time-lapse imaging of MSTO-211H-PDPN-luc-GFP with microscopic observations before and after NIR-PIT for 30 minutes.
